# Disparities in healthcare utilization by insurance status among patients with symptomatic peripheral artery disease

**DOI:** 10.1186/s12913-023-09862-1

**Published:** 2023-08-28

**Authors:** Brian Witrick, Corey A. Kalbaugh, Rachel Mayo, Brian Hendricks, Lu Shi

**Affiliations:** 1 West Virginia Clinical and Translational Sciences Institute, PO Box 9102, Morgantown, WV 26506-9102 USA; 2 Department of Epidemiology and Biostatistics, Indiana University School of Public Health, Bloomington, IN USA; 3 Department of Public Health Sciences, Clemson University, Clemson, SC USA; 4 Department of Epidemiology and Biostatistics, West Virginia University School of Public Health, Morgantown, WV USA

**Keywords:** Peripheral artery disease, PAD, Insurance status, Length of Stay, Healthcare utilization

## Abstract

**Background:**

Peripheral artery disease (PAD) is a common circulatory disorder associated with increased hospitalizations and significant health care-related expenditures. Among patients with PAD, insurance status is an important determinant of health care utilization, treatment of disease, and treatment outcomes. However, little is known about PAD-costs differences across different insurance providers. In this study we examined possible disparities in length of stay and total charge of inpatient hospitalizations among patients with PAD by insurance type.

**Methods:**

We conducted a cross-sectional analysis of length of stay and total charge by insurance provider for all hospitalizations for individuals with PAD in South Carolina (2010–2018). Cross-classified multilevel modeling was applied to account for the non-nested hierarchical structure of the data, with county and hospital included as random effects. Analyses were adjusted for patient age, race/ethnicity, county, year of admission, admission type, all-patient refined diagnostic groups, and Charlson comorbidity index.

**Results:**

Among 385,018 hospitalizations for individuals with PAD in South Carolina, the median length of stay was 4 days (IQR: 5) and the median total charge of hospitalization was $43,232 (IQR: $52,405). Length of stay and total charge varied significantly by insurance provider. Medicare patients had increased length of stay (IRR = 1.08, 95 CI%: 1.07, 1.09) and higher total charges (β: 0.012, 95% CI: 0.007, 0.178) than patients with private insurance. Medicaid patients also had increased length of stay (IRR = 1.26, 95% CI: 1.24,1.28) but had lower total charges (β: -0.022, 95% CI: -0.003. -0.015) than patients with private insurance.

**Conclusions:**

Insurance status was associated with inpatient length of stay and total charges in patients with PAD. It is essential that Medicare and Medicaid individuals with PAD receive proper management and care of their PAD, particularly in the primary care settings, to prevent hospitalizations and reduce the excess burden on these patients.

**Supplementary Information:**

The online version contains supplementary material available at 10.1186/s12913-023-09862-1.

## Background

Peripheral artery disease (PAD) is a common circulatory disorder that affects 8–12 million people in the United States [1, 2]. Individuals with PAD require numerous interactions with the health care system for care and are at a greater risk of functional impairment, limb loss, and death [3, 4]. Clinical management of PAD costs patients and the healthcare system an estimated 224–414 billion annually [5–7]. The majority of this economic burden stems from costs associated with frequent and recurring hospitalizations and repeat revascularizations [8–10].

Insurance status is a critical determinant of health care utilization, treatment of disease, and treatment outcomes [11]. People with PAD who are uninsured, underinsured, or have poor access to care, present with more severe vascular disease and have greater risk of unfavorable outcomes, including tissue loss or amputation [11–13]. Health care utilization and treatment costs increase with PAD severity, and patients with chronic limb threatening ischemia use more health care resources and incur higher costs than asymptomatic patients or those with claudication [10, 14]. Across insurance providers, individuals with PAD incur higher costs than individuals without PAD, particularly for those with public health insurance; however, little is known about PAD-costs differences across different insurance providers [7].

Approximately half (46%) of South Carolina residents are currently enrolled in a public insurance plan such as Medicare, Medicaid, military insurance plans, and other government- funded insurance plans [15]. The demand for public insurance in South Carolina will increase as the population of older adults is projected to double by 2030 [16] Compared to national estimates, adults in South Carolina have a higher burden of PAD risk factors, including smoking (18% vs. 16%), diabetes (14% vs. 11%), and cardiovascular disease (11% vs. 8%) [17]. Given the increased burden in risk factors, adults in South Carolina are likely at a greater risk of developing PAD and may have a higher demand for public health insurance in the future.

The purpose of this study is to examine disparities in length of stay and total charge of inpatient hospitalizations among patients with PAD by insurance type.

## Methods

### Study population

We obtained data for this cross-sectional study from the South Carolina Patient Encounter database (SCPED; 2010–2018). Detailed information about the SCPED and the data collection process has been described previously [18]. Briefly, the SCPED is managed by the South Carolina Revenue and Fiscal Affairs Office and serves as the central repository of all health and human services data in South Carolina, including claims and administrative data from all hospital inpatient within the state. This dataset has been used in previous studies to investigate risk factors of hospital outcomes and financial burdens in patients with a defined disease [19] [20]. This study was approved by the Clemson University Institutional Review Board (IRB2020-035).

Inclusion criteria for the study was all cause hospital admissions among patients 18 years or older with a confirmed diagnosis of PAD seen between January 2010 and December 2018. PAD status was determined using ICD-9-DM and ICD-10-DM codes (Supplementary Table 1) assigned in any position of the claim (primary or secondary). Hospital encounters with a discharge status of “expired” or “hospice”, or missing data for outcomes and covariates were excluded from the analysis to minimize event truncation.

### Data management

Covariates for the analysis included insurance status, age, race/ethnicity, county, year of admission, admission type, all-patient refined diagnostic groups (AP-DRG), and burden of pre-existing conditions measured by the Charlson comorbidity index (CCI). Insurance status was classified according to primary payor as Medicare, Medicaid, Self-Pay, Private, or Other (Worker’s compensation, indigent/charitable organization, and other Government insurance). Admission type was classified as urgent, emergent, elective, or other. Patient CCI indices were dichotomized such that patient with CCI ≥ 3 were classified as high burden, while CCI < 3 where moderate burden. County and hospital specific effects were adjusted for through inclusion of random effect terms. This approach provides an opportunity to adjust for county-level and hospital-level differences in care caused by external factors such as hospital policy and rurality in the analysis.

Outcomes measured included length of stay for inpatient admissions and total charges for all inpatient encounters among PAD patients. Length of stay was defined as the number of calendar days the patient was admitted as an inpatient. For this length of stay calculation, the day of admission was counted, but not the day of discharge; a patient admitted and discharged on the same day was assigned an length of stay = 1. Total charges included any charges the patient incurred while admitted, including but not limited to, room and board, pharmacy and lab, and professional fees. The total hospital charge is calculated by the chargemaster based on each procedure, service and good utilized during the specific encounter [21].

### Statistical analysis

Descriptive statistics were used for all demographic characteristics, comorbid conditions, and study outcomes. Normally distributed continuous variables are presented as mean ± standard deviation and statistical differences were identified using ANOVA. Non-normally distributed continuous variables are presented as median and interquartile range and statistical differences were identified using the Kruskal-Wallis test. Categorical variables are presented as proportions and chi-square testing was performed to identify differences across insurance groups.

Cross-classified multilevel regression modeling was implemented to account for the non-nested multilevel structure of the data [22]. Unlike in conventional multilevel analysis, cross-classified models consider that lower-level units are concurrently nested within two separate, non-nested hierarchies [23–25]. For example, in this study patients are simultaneously nested both in their counties and in the hospitals, but the hospital clusters are not necessarily nested within counties. Appropriately accounting for the structure of the data reduces omitted context bias and the likelihood of overstating the importance of included variables [22]. Cross-classified multilevel modeling has become increasingly popular in health care research to account for the multiple social and physical contexts in which patients reside [24, 26–28].

To find the appropriate estimation approaches for the outcome variables, we first investigated length of stay and total charges. Hospital length of stay was found to be positively skewed with an overdispersion of the variance. Therefore, negative binominal regression was utilized instead of Poisson regression. Total charges of patients with PAD were nonnegative and positively skewed by rare but extreme high-charge cases. To account for the violation of normality assumption, this study transformed the total charges by the logarithmic function [29]. Sensitivity analyses were conducted to control for discharge status and to account for the potential impact of discharge status on length of stay and total charge.

Data were analyzed using STATA 15 (Stata Corp, College Station, TX), and the threshold of statistical significance was set at p = 0.05. Both fixed effects and random-intercept effects for our multilevel regression were included with the hospital ID and the county set as level 2 random effects.

## Results

We identified 408,759 hospitalizations among 196,522 individuals with PAD in South Carolina between 2010 and 2018. After excluding 23,741 (5.8%) hospitalizations due to ineligible discharge status or missing variables, our analytic sample included 385,018 hospitalizations among 187,651 patients (Table 1). The average age of patients with PAD at hospitalization was 66.0 (SD 13.7) and more than half of hospitalizations were among male patients (56%). Nearly 70% of the hospitalizations were among Medicare patients, 14% among private insurance patients, and 7%, 5% and 5% among patients with Medicaid, Self-Pay, and Other, respectively. Overall, the majority of hospitalizations were for emergency admissions (57.1%) and were for patients considered high risk (56%).

**Table 1:** Overall Demographic and Hospitalization Measures by Insurance Provider

	Overall (N = 385,018)	Medicare (N = 267,255)	Medicaid (N = 27,672)	Self-Pay (N = 17,471)	Private Insurance (N = 52,057)	Other (N = 20,563)	P Value
**Mean Age**, **years (SD)**	66.0 (13.7)	70.8 (11.6)	52.2 (11.3)	50.4 (10.6)	57.6 (11.2)	56.4 (11.7)	< 0.000
**Race**							< 0.000
White	252,384 (65.6)	177,671 (66.5)	13,403 (48.4)	10,400 (59.5)	36,891 (70.9)	14,019 (68.2)	
Black	124,947 (32.5)	85,751 (32.1)	13,344 (48.2)	6,132 (35.1)	13,933 (26.8)	5,787 (28.1)	
Hispanic	1,863 (0.5)	706 (0.3)	308 (1.1)	305 (1.8)	268 (0.5)	276 (1.3)	
Asian	955 (0.3)	596 (0.2)	67 (0.2)	59 (0.3)	157 (0.3)	76 (0.4)	
American Indian	617 (0.2)	379 (0.1)	70 (0.3)	37 (0.2)	91 (0.2)	40 (0.2)	
Other	4,252 (1.1)	2,152 (0.8)	480 (1.7)	538 (3.1)	717 (1.4)	365 (1.8)	
**Female**	169,350 (44.0)	122,618 (45.9)	14,483 (52.3)	6,119 (35.0)	19,254 (37.0)	6,876 (33.4)	< 0.000
**Charlson Comorbidity Index**							< 0.000
Moderate	171,429 (44.5)	103,516 (38.7)	12,592 (45.5)	11,337 (64.9)	31,739 (61.0)	12,245 (59.6)	
High	213,589 (55.5)	163,739 (61.3)	15,080 (54.5)	6,134 (35.1)	20,318 (39.0)	8,318 (40.5)	
**Admission Type**							< 0.000
Emergency	219,993 (57.1)	153,688 (57.5)	17,224 (62.2)	11,593 (66.4)	25,915 (49.8)	11,573 (56.3)	
Urgent	83,592 (21.7)	56,907 (21.3)	5,473 (19.8)	3,510 (20.1)	13,026 (25.0)	4,676 (22.7)	
Elective	78,998 (20.5)	55,173 (20.6)	4,822 (17.4)	2,181 (12.5)	12,728 (24.5)	4,094 (19.9)	
Other	2,435 (0.6)	1,487 (0.6)	153 (0.6)	187 (1.1)	388 (0.8)	220 (1.1)	
**Admission Year**							< 0.000
2010	39,011 (10.1)	24,179 (9.1)	2,437 (8.8)	1,525 (8.7)	8,512 (16.4)	2,358 (11.5)	
2011	37,553 (9.8)	25,270 (9.5)	2,718 (9.1)	1,595 (11.2)	5,814 (10.5)	2,156 (9.8)	
2012	37,225 (9.7)	26,356 (9.9)	2,388 (8.6)	1,514 (8.7)	4,975 (9.6)	1,992 (9.7)	
2013	36,919 (9.6)	26,509 (9.9)	2,297 (8.3)	1,615 (9.2)	4,416 (8.5)	2,082 (10.1)	
2014	36,501 (9.5)	26,453 (9.9)	2,064 (7.5)	1,473 (8.4)	4,414 (8.5)	2,097 (10.2)	
2015	39,691 (10.3)	28,265 (10.6)	2,683 (9.7)	1,833 (10.5)	4,665 (9.0)	2,245 (10.9)	
2016	51,331 (13.3)	35,625 (13.3)	4,368 (15.8)	2,407 (13.8)	6,324 (12.2)	2,607 (12.7)	
2017	53,238 (13.8)	37,070 (13.9)	4,398 (15.9)	2,714 (15.5)	6,475 (12.4)	2,581 (12.6)	
2018	53,549 (13.9)	37,528 (14.0)	4,319 (15.6)	2,795 (16.0)	6,462 (12.4)	2,445 (11.9)	
**LOS, median (IQR)**	4.0 (5.0)	4.0 (5.0)	4.0 (6.0)	3.0 (4.0)	3.0 (4.0)	3.0 (5.0)	< 0.000
**Total Charge ($), median (IQR)**	43232.28 (52402.05)	41452.64 (50402.71)	40870.05 (52806.63)	45629.05 (56304.00)	51142.91 (56353.38)	51543.28 (56039.51)	< 0.000

**LOS = Length of stay**

Statistically significant differences across insurance status were observed for all demographic and hospitalization measures (Table 1). Medicare-associated hospitalizations were more likely to be older, with an average age of 70.8 (SD 11.6), male (54%), and have a higher burden due to comorbidities (56%). Hospitalizations of PAD patients with private insurance had an average age of 57.9 (SD 11.2), with 63% male, and only 39% having a high burden due to comorbidities. Hospitalizations associated with patients who self-paid were the youngest (50.4 ± 10.6) and only 35% were classified as high risk due to comorbidities. Medicaid-related hospitalizations were more likely to be to be female (52%) and be admitted due to an emergency (62%). All other hospitalizations had an average age of 56.4 (SD 11.7), were more likely to be male (67%) and White (68%).

**Table 2:** Multilevel regression of length of stay (Days)

	IRR (95% CI)	P value
**Insurance (Private = ref)**		
Medicare	1.078 (1.071, 1.093)	< 0.000
Medicaid	1.252 (1.243, 1.281)	< 0.000
Self-Pay	1.138 (1.132, 1.154)	< 0.000
Other	1.082 (1.069, 1.091)	< 0.000
**Age**	1.034 (1.024, 1.041)	< 0.000
**Female**	1.052 (1.041, 1.058)	< 0.000
**Race (White = ref)**		
Black	1.069 (1.062, 1.084)	< 0.000
Hispanic	1.001 (0.972, 1.039)	0.727
Asian	1.033 (0.991, 1.078)	0.125
American Indian	1.043 (0.979, 1.104)	0.249
Other	1.074 (1.046, 1.100)	< 0.000
**Charlson Comorbidity Index**		
High	1.242 (1.225, 1.253)	< 0.000
**Admission Type (Emergency = ref)**		
Urgent	0.981 (0.968, 0.984)	< 0.000
Elective	0.754 (0.742, 0.761)	< 0.000
Other/Unknown	1.100 (1.063, 1.139)	< 0.000

IRR = Incidence Rate Ratio

The length of stay for patients with PAD were significantly different based on insurance provider after adjusting for age, gender, race/ethnicity, CCI, admission type and year, county, and hospital (Table 2). Compared with patients with PAD with private insurance, patients with government-funded insurance had longer lengths of stay. Medicare patients had 7.8% (IRR = 1.078, 95 CI%: 1.071, 1.093) increased stays while Medicaid patients had a 25% (IRR = 1.252, 95% CI: 1.243, 1.281) increased stay. Patients with PAD who self-paid (IRR = 1.138, 95 CI%: 1.132, 1.154) or those with other types of insurance (IRR = 1.082, 95% CI:1.069, 1.091) also had significantly longer lengths of stay when compared to private insurance patients.

**Table 3:** Multilevel regression of total hospital charge ($)

	Regression Coefficient (95% CI)	P value
**Insurance (Private = ref)**		
Medicare	0.012 (0.007, 0.017)	< 0.000
Medicaid	-0.022 (-0.029, -0.015)	< 0.000
Self-Pay	-0.017 (-0.026, -0.009)	< 0.000
Other	0.007 (-0.001, 0.015)	0.080
**Age**	-0.012 (-0.001, -0.001)	< 0.000
**Female**	0.007 (0.004, 0.011)	< 0.000
**Race (White = ref)**		
Black	-0.026 (-0.030, − 0.022)	< 0.000
Hispanic	-0.020 (-0.042, 0.002)	0.080
Asian	-0.001 (-0.031, 0.030)	0.975
American Indian	-0.065 (-0.105, -0.026)	0.001
Other	-0.045 (-0.060, -0.030)	< 0.000
**Charlson Comorbidity Index**		
High	0.092 (0.089, 0.096)	< 0.000
**Admission Type (Emergency = ref)**		
Urgent	-0.102 (-0.106, -0.097)	< 0.000
Elective	-0.112 (-0.117, -0.107)	< 0.000
Other/Unknown	0.114 (0.093, 0.135)	< 0.000
**Length of Stay**	0.048 (0.045, 0.049)	< 0.000

Similarly, the total charges for patients with PAD also varied significantly by insurance provider (Table 3). After controlling for age, gender, race/ethnicity, CCI, AP-DRG, length of stay, admission type, year, county, and hospital, the total charges for patients with Medicare was 1.24% (β: 0.012, 95% CI: 0.007, 0.017) higher than patient with private insurance. Conversely, patients with Medicaid insurance had 2.18% (β: -0.022, 95% CI: -0.029, -0.015) lower total charges compared to patients with private insurance.

**Table 4:** Sensitivity analysis: length of stay (Days) and total hospital charge ($)

	LOS		Total Charge	
	IRR (95% CI)	P value	Regression Coefficient (95% CI)	P value
**Insurance (Private = ref)**				
Medicare	1.023 (1.009, 1.033)	< 0.000	-0.000 (-0.006, 0.004)	0.815
Medicaid	1.218 (1.209, 1.236)	< 0.000	-0.021 (-0.033, -0.015)	< 0.000
Self-Pay	1.185 (1.167, 1.195)	< 0.000	-0.006 (-0.014, 0.002)	0.153
Other	1.096 (1.072, 1.105)	< 0.000	0.009 (0.002, 0.017)	0.016
**Age**	0.973 (0.952, 0.978)	< 0.000	-0.027 (-0.028, -0.025)	< 0.000
**Female**	1.021 (1.007, 1.034)	< 0.000	-0.002 (-0.005, 0.001)	0.334
**Race (White = ref)**				
Black	1.061 (1.043, 1.067)	< 0.000	-0.033 (-0.036, -0.029)	< 0.000
Hispanic	1.053 (1.019, 1.088)	0.006	-0.014 (-0.036, 0.008)	0.225
Asian	1.054 (1.009, 1.101)	0.024	0.006 (-0.025, 0.036)	0.707
American Indian	1.056 (0.991, 1.117)	0.101	-0.062 (-0.101, 0.024)	0.001
Other	1.072 (1.005, 1.086)	< 0.000	-0.044 (-0.059, -0.030)	< 0.000
**Charlson Comorbidity Index**				
High	1.178 (1.172, 1.193)	< 0.000	0.078 (0.075, 0.082)	< 0.000
**Admission Type (Emergency = ref)**				
Urgent	0.998 (0.983, 1.005)	0.149	-0.097 (-0.101, 0.092)	< 0.000
Elective	0.792 (0.781, 0.806)	< 0.000	-0.099 (-0.104, -0.094)	< 0.000
Other/Unknown	1.091 (1.061, 1.134)	< 0.000	0.119 (0.098, 0.139)	< 0.000
**Discharge Category (Routine = ref)**				
SNF	2.144 (2.128, 2.163)	< 0.000	0.220 (0.214, 0.225)	< 0.000
HHA	1.552 (1.541, 1.568)	< 0.000	0.166 (0.162, 0.170)	< 0.000
Rehab	2.009 (1.971, 2.029)	< 0.000	0.293 (0.285, 0.301)	< 0.000
Long-term	2.301 (2.240, 2.356)	< 0.000	0.471 (0.453, 0.490)	< 0.000
Other	1.281 (1.267, 1.290)	< 0.000	0.023 (0.015, 0.031)	< 0.000
**Length of Stay**			0.045 (0.044, 0.045)	< 0.000

LOS = Length of stay; IRR = Incidence Rate Ratio; SNF = Skilled nursing facility; HHA = Home health agency

## Discussion

We found that the length of stay and total charges among patients with PAD varied by insurance status in our study of more than 385,000 hospitalizations in South Carolina. Patients with either Medicare or Medicaid insurance had longer lengths of stay when compared to patients with private insurance. Patients with Medicare insurance also had the highest overall charges. Despite a longer length of stay, patients with Medicaid insurance incurred lower overall charges compared to patients with private insurance. The longer length of stay observed in patients with PAD and public insurance reflects the excess burden experienced by these patients when compared to patients with private insurance.

Length of stay is a commonly used metric to assess patient quality of care. Prolonged hospitalizations put patients at risk for hospital acquired infections, readmissions, and mortality [30]. In our study, we found that individuals with PAD that have public insurance, particularly Medicaid, had longer lengths of stay. Thus, patients with PAD that use public insurance represent a high-risk group that may need targeted interventions to increase quality of care and reduce the potential risk associated with extended hospitalizations. Interventions that focus on proper identification and management of PAD in the primary care setting can both decrease the risk of hospitalization and shorten the length of stay if hospitalized [31]. Care coordination programs, which have successfully implemented interventions in patients with public insurance [32–34], should focus specifically on individuals with PAD to improve preventative care and decrease the burden experienced by these patients.

Patients with Medicare or Medicaid insurance in our study had longer lengths of stay than private insurance patients, a finding that is consistent with existing literature [35, 36]. Insurance status has a significant effect on patients with PAD underdoing a lower extremity bypass intervention [36]. Patients with private insurance spend significantly less time in the hospital than patients with Medicare or Medicaid insurance [36]. Our study expands upon these findings by not limiting our population to hospitalizations occurring after a vascular intervention, which occur in only half of PAD-related hospitalizations [10]. Through the inclusion of all-cause hospitalizations, regardless of revascularization, we are better able to understand the role that insurance has on length of stay in patients with PAD.

Variability in hospital charges by insurance has been shown before [37]. Our study extends this literature to populations with PAD in South Carolina. Medicaid patients had the lowest charges, followed by privately insured patients, and patients with Medicare. Reimbursement rates, which determine how much a patient or insurance company must pay for a given hospitalization encounter, are known to vary based on insurance status [38]. The payment rates for private insurance are 70% greater than Medicare or Medicaid [39]. However, within the same hospital, charges are assumed to be constant across payers [37, 40]. Hospitals may be inflating charges based on insurance status despite providing similar care. While our study was not designed to test this hypothesis, a future study should investigate if hospitals are differentially inflating charges based on insurance.

Insurance providers have policies that have implications for health care utilization. As an example, to qualify for coverage of post-acute care in a skilled nursing facility, a patient enrolled in the original Medicare program must be admitted in an inpatient hospital for at least three consecutive days. Requiring patients to be admitted for three days can unnecessarily lengthen stays in inpatient facilities and increases the total charges [41, 42]. Therefore, the increased total hospital charges by PAD patients with Medicare insurance found in our study could be partly due to the effects of the Medicare “three-day” policy [43]. To test the potential impact of this Medicare policy, we controlled for discharge status in the sensitivity analysis. The adjusted length of stay for individuals with Medicare was only 2% greater than individuals with private insurance and there was no difference in total charges between the groups (Table 4). The findings suggests that Medicare’s “three-day” policy has an impact on health care utilization and should be reconsidered.

An important strength of this study is the comprehensive, population-based dataset that includes all payer data throughout the entire state. The dataset spans multiple years and allows for the ability to continuously obtain data in South Carolina, even if individuals move within the state, change insurance status, or change employment. The breadth and continuity of the dataset allows for understanding and generalizations across the state, which will aid in policy decision making, particularly preventative measures and Medicaid reimbursement. Furthermore, the South Carolina population is racially diverse, with twice the proportion of Black individuals as compared to national estimates (26% vs. 13%) [17]. PAD disproportionally affects Black individuals and the racial diversity in the South Carolina population increases our understanding of the total impact of PAD on these individuals.

This study has several limitations that should be noted. First, administrative claims data are collected for billing purposes only. Inaccurate diagnostic coding in regard to clinical diagnosis or procedures and variations in coding practices between hospitals and medical practices is possible [44, 45]. Further, assessing PAD severity using claims data is difficult because of frequent use of generic coding that does not indicate the anatomic severity of disease or the location of that disease [46]. Our inability to control for undiagnosed medical conditions and important clinical factors, such as anatomical disease or symptomatic severity, may lead to residual confounding. Second, our patients with public insurance had more comorbid conditions at time of admission than patients with other insurance providers. It is possible that previous poor management of those risk factors may increase hospitalization length. Unfortunately, due to cross-sectional nature of the data, we are unable to follow patients over time and cannot account for previous hospitalizations. While we control for comorbidity differences by using the CCI, we are unable to control for the effect of poor management prior to the hospitalization or recurrent hospitalizations. Third, Medicare hospitalizations account for the majority of hospitalizations in this study. Nonetheless, our robust sample size for each insurance provider allowed us to have precise estimates for both outcomes and we do not believe this to be a limitation that would change our conclusions. Fourth, insurance status was limited to only the primary payor and does not differentiate individuals who might have more than one type of insurance or specifically identify which insurance plan patients had. Patients who are dually enrolled in Medicare and Medicaid have a higher burden of comorbidities and healthcare utilization than other patients [47]. Additionally, patients enrolled in Medicare advantage utilize healthcare services differently than traditional Medicare enrollees [48]. Further research should investigate dual Medicare-Medicaid eligibility and specific Medicare plans. Finally, it is important to note that our study utilized total hospital charges. Previous research on healthcare costs have operationalized costs in several different ways, including total hospital charges, cost-to-charge ratios, and reimbursements, which can be make it difficult to directly compare findings across studies [49].

## Conclusion

Insurance status was significantly associated with inpatient length of stay and total charges in patients hospitalized with peripheral artery disease. Patients with peripheral artery disease and public insurance may experience excess risks associated with long hospital stays, particularly hospital-acquired infections. Thus, Medicare and Medicaid individuals with PAD must receive proper management and care of their PAD, particularly in the primary care settings, to prevent hospitalizations and reduce the excess burden on these patients.

### Electronic supplementary material

Below is the link to the electronic supplementary material.

Supplementary Material 1

## Data Availability

The datasets analyzed for this study obtained from the South Carolina Department of Revenue and Fiscal Affairs Office and are available from the authors with the permission of the South Carolina Revenue and Fiscal Affairs Office. Please contact Brian Witrick.

## List of abbreviations

PAD: Peripheral artery disease.
SC PED: South Carolina patient encounter database.
AP-DRG: All patient refined diagnostic groups.
CCI: Charlson comorbidity index.
LOS: Length of stay.
IRR: Incidence rate ratio.
SNF: Skilled nursing facility.
HHA: Home health agency.
