# Supplementary material for: Disparities in healthcare utilization by insurance status among patients with symptomatic peripheral artery disease
Source: BMC Health Serv Res. 2023 Aug 28;23:913. doi: 10.1186/s12913-023-09862-1 (PMC10463334; doi:10.1186/s12913-023-09862-1)
Supplement: Supplementary file 1 — Supplementary Material 1 [file 12913_2023_9862_MOESM1_ESM.docx]

Supplementary Material

# Supplementary Data

ICD-9-CM and ICD-10-CM Diagnosis Codes for Peripheral Artery Disease

| **Condition** | **ICD-9 Codes** | **ICD-10 Codes** |
| --- | --- | --- |
| Critical Limb Ischemia | 440.22, 440.23, 440.24, 707.10,  707.11, 707.12, 707.13, 707.14,  707.15, 707.16, 707.17, 707.18,  707.19, 785.4 | I70.229, I70.25, I70.269, L97.909, L97.109, L97.209, L97.309, L97.409, L97.509, L97.809, I96 |
| Peripheral Artery Disease | 440.2, 440.20, 440.21, 440.22, 440.23, 440.24, 440.29, 440.3, 440.0, 440.30, 440.31, 440.32, 440.9, 249.7, 249.71, 250.7, 250.71, 250.72, 250.73, 443.1,  443.81, 443.9, 444.22, 444.81, 785.4 | I70.209, I70.219, I70.229, I70.25, I70.269, I70.299, I70.0, I70.399, I70.499, I70.599, I70.90, I70.91, E08.51, E09.51, E13.59, E08.51, E08.65, E09.51, E11.51, E10.51, E11.51, E11.65, E10.51, E10.65, I73.1, I79.8, I73.9, I74.5, I74.3, I96 |
